# Platelet-cytokine Complex Suppresses Tumour Growth by Exploiting Intratumoural Thrombin-dependent Platelet Aggregation

**DOI:** 10.1038/srep25077

**Published:** 2016-04-27

**Authors:** Yu-Tung Li, Tomoyuki Nishikawa, Yasufumi Kaneda

**Affiliations:** 1 Division of Gene Therapy Science, Graduate School of Medicine, Osaka University, Osaka, Japan

## Abstract

Tumours constitute unique microenvironments where various blood cells and factors are exposed as a result of leaky vasculature. In the present study, we report that thrombin enrichment in B16F10 melanoma led to platelet aggregation, and this property was exploited to administer an anticancer cytokine, interferon-gamma induced protein 10 (IP10), through the formation of a platelet-IP10 complex. When intravenously infused, the complex reached platelet microaggregates in the tumour. The responses induced by the complex were solely immune-mediated, and tumour cytotoxicity was not observed. The complex suppressed the growth of mouse melanoma *in vivo*, while both platelets and the complex suppressed the accumulation of FoxP3^+^ regulatory T cells in the tumour. These results demonstrated that thrombin-dependent platelet aggregation in B16F10 tumours defines platelets as a vector to deliver anticancer cytokines and provide specific treatment benefits.

Cancer is a heterogeneous disease with a unique microenvironment where stromal cells in solid tumours are modulated to support tumour growth and progression^1^. An angiogenic environment with vascular enrichment frequently develops for sustainable nourishment^2^. However, a dynamic plasticity exists between neoangiogenesis and regression in the tumour vasculature^3^. In addition, a rapidly growing tumour mass and the presence of a cocktail of growth factors and cytokines contributes to a leaky local vasculature, exposing various blood components to the tumour tissue^4^. The accumulating knowledge of cancer biology has paved the way for the development of alternative therapies to traditional chemotherapy, which is frequently accompanied by severe side effects. For example, understanding the molecular profiles of tumour cells has led to several new approaches, such as drug-coupled monoclonal antibodies against tumour-associated antigens^5^ and adoptive T cell therapy^6^.

Platelets are small blood cells that perform critical functions in haemostasis. Under physiological conditions, platelets are maintained at the resting state and are prevented from contacting subendothelial components in the endothelium. In the event of injury, platelets are exposed to the subendothelial collagen surface and become activated through glycoprotein VI engagement^7^. In addition, the tissue factor pathway is induced upon contact between factor VIIa and subendothelial tissue factor, likely contributing to platelet activation^8^. The factors involved in coagulation support growth, angiogenesis and metastasis, either by direct signalling or platelet-mediated mechanisms in malignant tissues^9^,^10^,^11^. Thrombin, a critical component in the coagulation cascade, relays an activating signal to platelets through the cleavage of protease-activated receptors (PARs) to trigger the downstream aggregation and release of granular contents^12^. In some cancer lines, thrombin is actively secreted from tumour cells^13^, and thrombin inhibition by argatroban, a direct thrombin inhibitor (DTI), reduces malignancy^14^,^15^. Matsumura *et al.* recognized a persistent microscopic haemorrhage in the tumour and introduced the concept of cancer stromal targeting (CAST) therapy, which describes a cytotoxic immunoconjugate entrapped within the tumour stroma, enabling the prolonged release of an anticancer drug as a passive targeting strategy^16^. For example, a drug-conjugated antibody against fibrin, specifically formed within a tumour, was effective in tumour suppression^17^. The efficacy of platelets containing inactivated Sendai virus particles, which colocalize to intratumoural fibrin clots, for cancer treatment has also been reported^18^.

Cytokines represent a class of protein therapeutics with clinical uses to treat cancer. However, administration through an intravenous route involves toxicity, reflecting peripheral bioactivity^19^,^20^. Currently, this issue had been managed by the modulation of the administration route, schedule and dose. An alternative strategy is to concentrate and enhance the efficiency of the cytokine in the tumour using a delivery vehicle. Interferon-gamma induced protein 10 (IP10), also known as CXCL10, induces the migration of immune cells towards tumours via chemotaxis mediated by CXCR3 receptor interactions. As a downstream effector of interferon-gamma (IFN-γ), the antitumour activity of IP10 has been previously demonstrated in several cancer types, such as breast, B Burkitt lymphoma and myeloma^21^,^22^,^23^. The interaction of this chemokine with endothelial cells leads to angiostatic responses^24^,^25^.

Targetable stromal components in the tumour microenvironment, such as matrix metalloproteinases (MMPs), are emerging^26^, and the expansion of this knowledge and novel uses of the identified targets are expected to contribute to future cancer therapy development. In the present study, we hypothesized that intratumoural thrombin preferentially activates and retains infiltrating platelets in thrombotic clots, a property that might enable platelets to function as vectors for the transport of cytokines into the tumour. The antitumour activity of the model cytokine, IP10, embedded in platelets was subsequently examined using the B16F10 melanoma model. This model offers an advantage to investigate any immunological response of interest, as B16F10 cells were implanted in immunocompetent C57BL/6 mice.

## Results

### Elevated thrombin and aggregated platelets were detected in tumours

**Figure 1:**
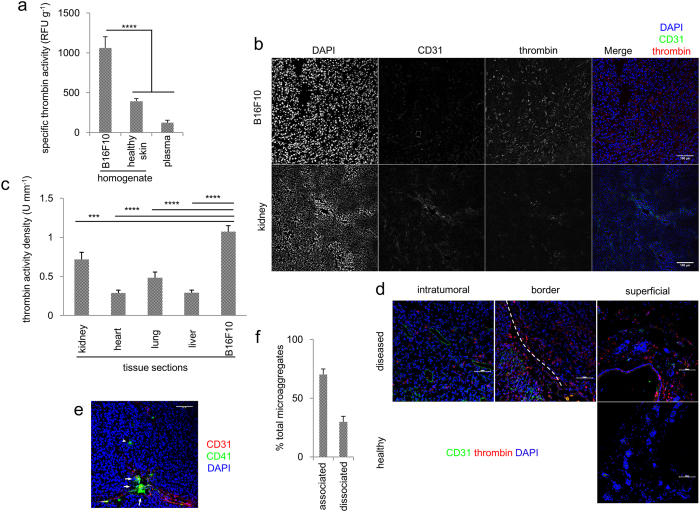
Active thrombin is elevated in B16F10 melanoma and platelet aggregates were detected in the vasculature proximity. (**a**) Plasma, freshly homogenized tissues of B16F10 and skin were measured for thrombin activity using SensoLyte assay kits. n = 3 mice. Error bars, SD. ****p < 0.001. One-way ANOVA followed by Dunnett’s test. (**b**) Sections from whole B16F10 tumours (upper panel) or kidney samples (lower panel) were stained for cleaved thrombin (red) and CD31 (green) and examined using a confocal microscope. Scale bars = 100 μm. (**c**) Thrombin activities of tissue sections from tumours and various normal organs were determined and normalized to the tissue volume. n = 4 mice. ***p < 0.005, ****p < 0.001. One-way ANOVA followed by Dunnett’s test. (**d**) Thrombin distribution in the surrounding tissues of B16F10 (upper panel) and unaffected skin (lower panel). The white dotted line indicates the tumour border. Scale bars = 100 μm. (**e**) Relative localization between vasculature (CD31, red) and platelet aggregates (CD41, green) in the B16F10 tumour. The arrowhead indicates a vessel-dissociated aggregate, and the arrows indicate vessel-associated aggregates. Scale bar = 100 μm. (**f**) In total, 174 clusters of intratumoural platelet aggregates from 7 tumours were examined for spatial relationship with the vasculature. Error bars, SEM.

After more than three weeks, transplanted B16F10 xenografts often show a fibrotic surface accompanied by occasional bleeding. Thus, we expected to detect the presence of active thrombin, a critical factor in coagulation initiated by tissue damage, in the tumour. The thrombin activities of plasma, homogenates of B16F10 melanoma or skin from healthy mice were significantly higher in the tumour. In contrast, the abundant prothrombin in plasma remained inactive, and minimal prothrombin activity was detected (Fig. 1a). To better observe the thrombin distribution in tumours, frozen sections of B16F10 melanoma and kidney were prepared for confocal microscopy. A readily observable thrombin signal was detected in B16F10 tissues but only rarely detected in kidney tissues (Fig. 1b). Importantly, the majority of thrombin signals were observed in extracellular compartments within the tumour mass, suggesting that the intratumoural environment facilitates extracellular thrombin production. This effect is unlikely B16F10-specific, as a similar localization was observed in PC3 human prostate cancer xenografts (Supplementary Fig. S1). Thrombin activity was enriched in B16F10 compared with the normal tissues of other organs (Fig. 1c). Unexpectedly, thrombin enrichment was not localized within the tumour where a dynamic vasculature is present but extended across the tumour border to superficial skin. This feature suggests that the tumour imposed damaging stress to surrounding normal tissues (Fig. 1d).

As thrombin is a potent platelet agonist, we speculated that platelets might be activated to form aggregates. As expected, numerous platelet microaggregates were observed in clusters within the tumour. A total of 174 microaggregate clusters were examined, and the majority of these clusters were associated with the vasculature, indicating vessel-proximal activation and suggesting the presence of damaged vasculature (Fig. 1e,f).

Next, we examined whether tumour cells alone are sufficient to activate platelets. The platelets were incubated with B16F10 cells *in vitro* and examined for CD62P expression and fibrinogen binding. CD62P is an α-granule protein that translocates to the plasma membrane upon activation. In addition, activated platelets exhibit a higher affinity to fibrinogen as a result of inside-out signalling. No direct activation or thrombin-producing enzymatic activity was observed in tumour cells, as these cells failed to activate platelets, regardless of the presence of plasma prothrombin. The platelets were neither activated using conditioned medium nor a secreted agonist (Supplementary Figs S2 and S3). Despite the incapability of B16F10 to activate platelets *in vitro*, the affinity between B16F10 and platelets changed with the activation of these thrombocytes. B16F10 cells were incubated with resting or activated platelets, and the bound platelets were detected using flow cytometry. Stronger binding was observed with activated platelets than at the resting state (Supplementary Fig. S2). This change in affinity suggested post-activation deposition, facilitating the formation of tumour-platelet complexes (Fig. 1e, arrowhead). Taken together, these results suggested an *in vivo* tumour microenvironment, rather than stand-alone tumour cells, is required for thrombin enrichment and platelet activation in the B16F10 model.

### Formation of the IP10-in-platelet complex

**Figure 2:**
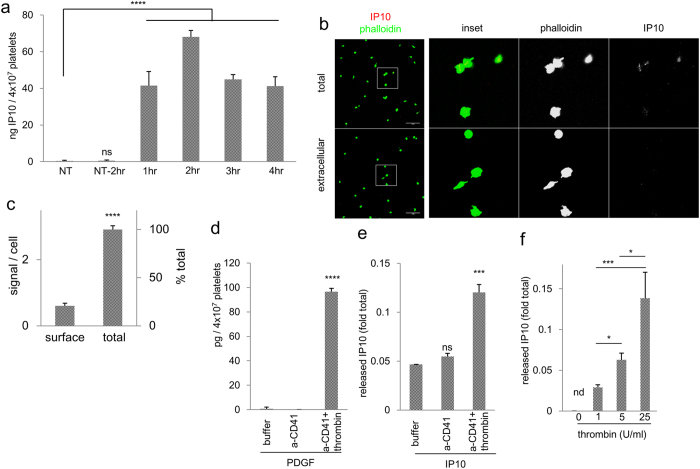
Incorporation and release of IP10 in platelets. (**a**) Fresh platelets were incubated with rm-IP10 in mTyrode’s buffer at 37 °C. The platelets were isolated at time points and lysates were measured for IP10 by ELISA. n = 3. NT, no treatment; NT-2 hr, NT incubated at 37 °C for 2 hr. (**b**) Platelet-IP10 complexes were stained for IP10 (red) with or without permeabilization and counterstained using phalloidin (green). Scale bars = 20 μm. 100× objectives. (**c**) More than 100 complexes were examined, and the IP10 signals were enumerated. ****p < 0.0001. Student’s *t*-test. (**d**,**e**) Complexes were treated with buffer, anti-CD41, or anti-CD41 plus thrombin. The released contents were assayed for PDGF (**d**) and IP10 (**e**) using ELISA. n = 3. (**f**) Complexes were stimulated with an increasing concentration of thrombin, and assayed for IP10 release using ELISA. n = 3. Error bars, SEM. nd, not detected, *p < 0.05, ***p < 0.005. One-way ANOVA followed by Dunnett’s test (for (**a**,**d**,**e**)) or Tukey’s test (**f**) was performed.

As activated platelets form clusters and remain in the tumour, if an anti-cancer protein is incorporated into the platelet granules, platelets have the potential to locally release the protein drug when stimulated by the tumour microenvironment. Platelets physiologically endocytose ambient proteins into α-granules^27^. We have previously succeeded in employing this process to incorporate Haemagglutinating Virus of Japan Envelope (HVJ-E) into platelets^18^. We employed IP10 as a model therapeutic protein and antitumour chemokine^21^,^22^,^23^ to prepare the platelet-IP10 complex. IP10 is not expressed in native platelets, and upon incubation with platelets, recombinant IP10 incorporation peaked after 2 hours (Fig. 2a). To confirm successful incorporation, the platelet-IP10 complex was treated with either Triton X-100 or buffer to differentiate total and surface IP10 molecules, and the majority (approximately 80%) of IP10 was detected inside platelets (Fig. 2b,c). Further characterization showed the colocalization of IP10 with PF4, an abundant protein in α-granules, but not with CD63, a marker protein for dense granules, confirming the presence of IP10 within α-granules (Supplementary Figs S4 and S5). In subsequent experiments, the platelet-IP10 complex was prepared after a 2-hour incubation.

To investigate whether the incorporated IP10 could be released upon activation, the complex was stimulated with thrombin, and the released proteins were analysed. Thrombin rapidly stimulated platelets in 10 minutes based on the surface expression of CD62P (Supplementary Fig. S2). Both PDGF, an intrinsic protein in α-granules, and IP10 were released after thrombin stimulation, and IP10 release is a dose-dependent response mediated by thrombin (Fig. 2d–f). In summary, the platelet-IP10 complex was successfully formed and IP10 was released by thrombin stimulation.

### Formation of platelet microaggregates where the platelet-IP10 complex reaches B16F10 tumours as a thrombin-dependent process

**Figure 3:**
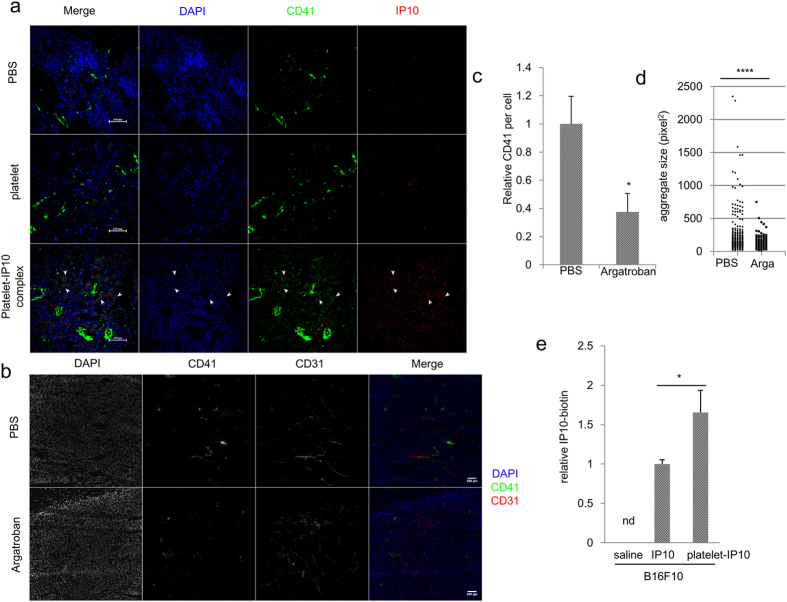
Tumour targeting of the platelet-IP10 complex. (**a**) Platelet or platelet-IP10 complex or PBS was intravenously injected into B16F10-bearing mice through the tail vein for three consecutive days. The tumour sections were stained for platelet aggregates (CD41, green) and IP10 (red). Arrowheads show examples of aggregation of the platelet-IP10 complex (yellow). Scale bars, 100 μm. (**b**,**c**) Tumour-bearing mice were peritoneally injected with argatroban or PBS. PFA perfused tumour tissues were stained for CD31 (red) and CD41 (green). 10× objective. n = 4 mice. Scale bar = 100 μm. *p < 0.05. Student’s *t*-test. (**d**) PKH67-labelled platelets were injected with argatroban (arga) or PBS to pre-conditioned tumours. The sizes of the platelet microaggregates in PBS (n = 237) and argatroban (n = 113)-treated tumours were measured. (**e**) The platelet-biotinylated IP10 complex was constructed, at 2 hours after treatment with a single intravenous dose, the tumours were harvested and measured for intratumoural biotinylated IP10 using ELISA. n = 4–9 tumours per group. nd, not detected, *p < 0.05, ****p < 0.001. Mann-Whitney *U*-test for (**d**,**e**). Error bars, SEM.

Platelet aggregation in tumours prompted us to investigate whether exogenously injected platelets could reach the tumour and show the same aggregation phenotype. When platelets were intravenously injected into mice bearing tumour xenografts, the IP10 staining signal was basal and comparable with that of the PBS control, consistent with the absence of IP10 in native platelets as shown in Fig. 2a. IP10 was more abundantly distributed in tumours harbouring the injected platelet-IP10 complex (Fig. 3a). Notably, the injected platelet complex (yellow) accumulated in weakly stained CD41^+^ regions, likely corresponding to younger and developing platelet aggregates, and deposited onto the tumour cell (Fig. 3a and Supplementary Fig. S6). These results showed that the injected complex was capable of reaching the tumour and becoming activated *in situ*. The dependency of platelet microaggregation on active thrombin in B16F10 tumours was subsequently studied. After 8 consecutive daily intraperitoneal doses of either argatroban or PBS, tumour-bearing mice were PFA-perfused and examined for intratumoural platelet microaggregates. Argatroban is a univalent direct thrombin inhibitor (DTI) that functions by directly binding to the active site of thrombin to prevent the activity of this protein^28^. Argatroban treatment reduced platelet microaggregation in the tumour, shown as a lower platelet-specific CD41 signal normalized to the nuclei number (Fig. 3b,c). Further examination of the distribution of the injected platelet-IP10 complex indicated co-localization with intratumoural thrombin (Supplementary Fig. S7). As platelets were solely found activated near the vessel, we hypothesized that vessel rupture exposes circulating platelets to the thrombin-rich tumour microenvironment, leading to platelet activation and aggregation. To assess this model, PKH67-labelled platelets were directly exposed to the tumour microenvironment to mimic vessel rupture in the presence of argatroban or PBS. The numerous platelet microaggregates thus formed were scored for size. As argatroban suppressed platelet aggregation and significantly reduced the size of the microaggregates, intratumoural platelet microaggregation is a thrombin-dependent process (Fig. 3d).

To further verify whether the platelet-IP10 complex delivers IP10 to the tumour as shown in Fig. 3a, a single dose of biotin-labelled IP10 was intravenously injected either alone or in the platelet complex into B16F10 tumour-bearing mice and circulation was permitted for 2 hours. ELISA measurement for the labelled IP10 in the tumour revealed that more IP10 accumulated in the tumour tissues when IP10 was incorporated within platelets (Fig. 3e). Taken together, these findings support an essential role for intratumoural thrombin in the formation of intratumoural platelet microaggregates, where the platelet vector facilitates tumour access to the cargo protein.

### Effects of the platelet-IP10 complex on B16F10 melanoma growth *in vivo*

**Figure 4:**
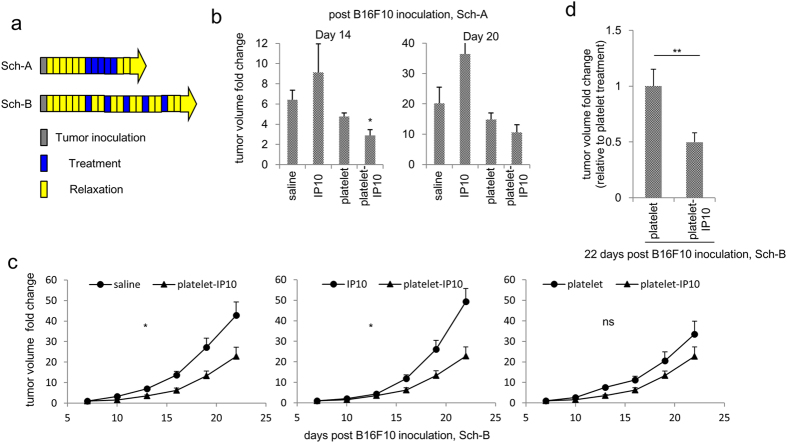
Antitumour effects of platelet-IP10 complex *in vivo*. (**a**) Schematic representation of the treatment schedules (Sch). Each block represents one daily unit, and the arrows represent ongoing monitoring. (**b**) The volume of the B16F10 tumour at 14 and 20 days post-tumour inoculation. n = 5 mice per group. (**c**) Tumour growth time course study on mice bearing 7-day old subcutaneous B16F10 tumour received indicated intravenous treatments. n = 10–12 mice per group. Error bars, SEM. *p < 0.05. One-way ANOVA was followed by Dunnett’s test in (**b**,**c**). (**d**) The tumour volumes in the platelet-IP10 treatment group were compared with those in the platelet control group at 22 days post-tumour inoculation. The data are pooled from (**c**) and two other independent experiments. n = 21–22 mice per group. **p < 0.01. Mann-Whitney *U*-test.

To examine the antitumour potency of the platelet-IP10 complex, one week after subcutaneous tumour inoculation, the mice received daily treatment for 5 days (Fig. 4a, Schedule A). Tumour growth was significantly suppressed after platelet-IP10 complex treatment compared with saline and platelet controls at one week after treatment (Fig. 4b). When the same treatments were dispersed over a longer period (Fig. 4a, Schedule B), platelet-IP10 complex treatment similarly resulted in significant tumour growth reduction compared with saline and IP10 controls (Fig. 4c). It is evident that platelets carrying IP10 exhibited better antitumour activity than the vector alone (Fig. 4d).

### Immune responses triggered by the platelet-IP10 complex *in vivo*

A potential issue with the use of platelets is the release of angiogenic factors, such as vascular endothelial growth factor (VEGF), upon activation, which is undesirable for treating cancer. When exposed to platelet releasate, human aortic endothelial cells (HAEC) proliferated more than two-fold faster than control *in vitro*, while 0.5 μg/ml IP10 exhibited no angiostatic effect. However, in tumour tissues, the enhancement of endothelial cell proliferation using this platelet vector system was not prominent *in vivo* (Supplementary Fig. S8). Moreover, major cytotoxicity was not associated with the platelet-IP10 complex or with either component alone (Supplementary Fig. S9). Therefore, we focused on immune responses *in vivo* to investigate the mechanism underlying the antitumour effects of the platelet-IP10 complex.

**Figure 5:**
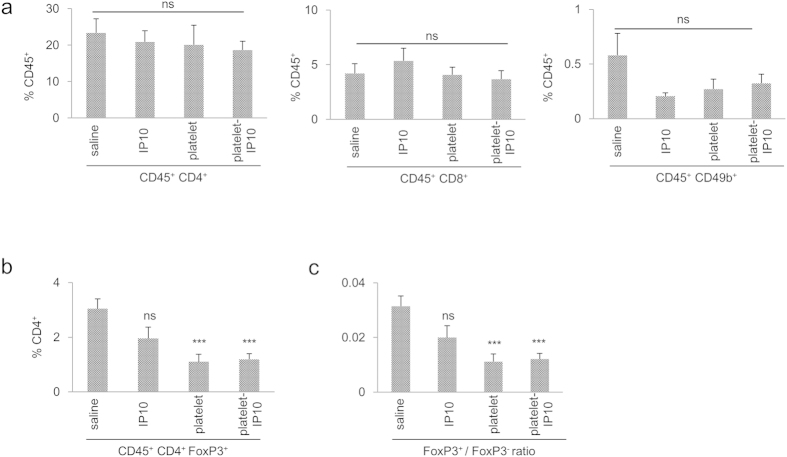
Cellular immune responses induced by the platelet-IP10 complex *in vivo*. The mice were inoculated with B16F10 cells and treated following Schedule A, as described in Fig. 4a. The excised tumours were digested with 0.5% collagenase containing 2% FBS until dissociation. Intracellular immune cells were stained for CD4, CD8 and CD49b (**a**) and FoxP3 (**b**) gated at CD45^+^ and analysed using flow cytometry. In (**c**), the ratio of FoxP3^+^ to FoxP3^−^ cells is shown. n = 4–5 tumours. ns, not significant, ***p < 0.005. One-way ANOVA followed by Dunnett’s test was used throughout this figure.

**Figure 6:**
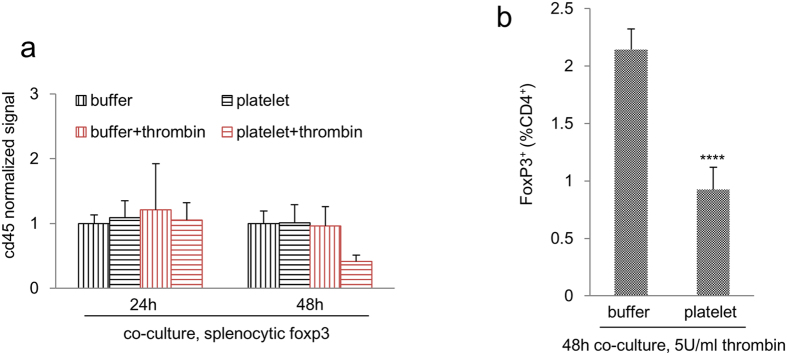
Platelet modulates FoxP3 expression. (**a**) Splenocytes were co-cultured with buffer or platelet for 24 (n = 7–10) or 48 hours (n = 3–7) in the presence or absence of thrombin, and foxp3 expression was analysed using qRT-PCR. (**b**) Splenocytes were treated as in (**a**) for 48 hours in the presence of thrombin, FoxP3 expression was analysed using flow cytometry. n = 9–10. Error bars, SEM. ****p < 0.0005. Student’s *t*-test.

The number of intratumoural T cells and NK cells was assessed using flow cytometry. There was no obvious alterations in the numbers of intratumoural CD4^+^ or CD8^+^ T cells or NK cells between treatments (Fig. 5a). However, the CD4^+^ FoxP3^+^ population was considerably reduced in platelet- and platelet-IP10 complex-treated mice (Fig. 5b), shifting the balance between regulatory and effector CD4^+^ T cells (FoxP3^+^/FoxP3^−^) away from immunosuppression (Fig. 5c and Supplementary Figs S10 and S11). Indeed, a single dose of platelet-IP10 complex was sufficient to trigger a reduction in regulatory T cells (Supplementary Fig. S12). This modulation in regulatory T cells was likely mediated by the platelet vector. Indeed, when splenocytes were co-cultured with platelets under thrombin activation for 48 hours, we observed a noticeable decrease in the FoxP3 transcript and a significant reduction in FoxP3 protein (Fig. 6a,b and Supplementary Fig. S13). As the transcription factor FoxP3 is crucial for the immunosuppressive activities of regulatory T cells^29^, this platelet-mediated suppression of FoxP3 expression represented a mechanism by which the platelet complex exerts antitumour effects.

## Discussion

Thrombocytopenia is a frequent manifestation following common cancer treatments, such as systemic chemotherapy, and platelet transfusion is often necessary to prevent uncontrolled bleeding^30^,^31^. While the current practice of platelet transfusion serves a prophylactic purpose, the results of the present study implied that this measure might be explorative with regard to integration with cytokine therapy. The current understanding of the roles of platelets in cancer is multifaceted, with overwhelming reports suggesting that platelets facilitate metastasis^32^,^33^,^34^,^35^,^36^,^37^. As tumour growth enhancement by the platelet vector was not evident *per se*, the precise role of platelets in the growth of various primary tumours might require further investigation. However, there is accumulating evidence for the pro-inflammatory functions of platelets in various disease conditions^38^,^39^. In the present study, we affirmed that platelets induce an antitumour response early in progression through the downregulation of FoxP3^+^ regulatory T cells. In atherosclerosis and asthma, platelets suppress regulatory T cell recruitment and accelerate disease progression mediated by platelet CD40L^38^,^40^,^41^. Blocking CD40-CD40L interactions unleashed suppression to regulatory T cell expansion^42^, and the results of the co-culture experiment in the present study suggested that the addition of platelets suppressed the expansion of the regulatory T cell population. Activated platelets express CD40L on the cell surface and release platelet microparticles (PMPs) harbouring functional membrane-bound CD40L^43^. The classical CD40-CD40L interaction transduces a downstream signal through adaptor molecules known as tumour-necrosis-factor-receptor-associated factors (TRAFs). Intriguingly, the disruption of the CD40-TRAF2/3/5 interaction in MHC-II expressing cells, including activated T cells, increased the number of regulatory T cells underlying atherosclerotic plaques^44^. Thus, this result implies that the platelet-CD40L-CD40-TRAF2/3/5 axis in MHC-II-expressing antigen presenting cells is important to suppress regulatory T cell recruitment. In addition, the soluble version of CD40L, sCD40L, might serve as a potential soluble modulator in cancer. To our knowledge, this study is the first to show that this platelet-mediated function could be utilized in cancer treatment.

Platelet granules contain a variety of immune and growth regulators, and there are concerns regarding potential undesired effects. For growth modulators that favour tumour cell growth, including CXCL1^45^,^46^ and CXCL12^47^, the results excluded any major proliferative effects under the platelet vector context (Supplementary Fig. S9). Moreover, a mixture of both angiogenic and antiangiogenic factors are released by activated platelets. For example, VEGF and various CXCR2 ligands^48^ contained in the α-granules promote endothelial growth. However, platelets contain abundant TSP-1^49^,^50^,^51^, PF4^52^,^53^,^54^ and the more potent variant CXCL4L1^55^, which suppress endothelial cell growth and migration by various mechanisms, including apoptosis induction, signalling interference and MAPK pathway inhibition. As a result of the antagonism between the two groups of factors, a net effect on tumour angiogenesis was absent with the platelet vector, as the application did not significantly alter the number of intratumoural endothelial cells *in vivo* (Supplementary Fig. S8). In addition, it has been proposed that the granular proteins in the platelets are organized such that different subsets are released in response to specific molecular engagement^56^. As cancers of different types express distinct molecular profiles and thus might differentially interact with platelets, the roles of platelets might vary between cancers. When cytokines are administered as a platelet complex, the biologically properties of the platelets are further altered and implications in tumour biology should be re-evaluated.

Apart from directly participating in malignancy progression events, thrombin is involved in inducing venous thromboembolism (VTE), where a venous thrombus breaks the blood flow, and depending on the location of VTE, this condition can be life threatening. Anticoagulants, such as heparin and vitamin K antagonist, which target various components in the coagulation cascade, are often prescribed to address this condition^57^,^58^. We showed that the specific inhibition of thrombin by argatroban reduced but did not abolish intratumoural platelet aggregates. It is therefore likely that the platelet vector retains the capability to reach the tumour using anticoagulants. Simultaneous uses of an anticoagulant and the platelet vector might eliminate the stray incorporation into peripheral thrombi and more effectively drain the protein drug cargo into the tumour. Further investigation is required to verify whether this measure optimizes the use of platelet vector.

Although the frequent occurrence of platelet microaggregates indicates activation in B16F10 tumours, direct platelet activation by B16F10 cells was not observed *in vitro*, consistent with a previous report showing that platelet activation by B16F10 *in vitro* was a heparin-mediated artefact^59^. For intratumoural platelet activation, the results of the present inhibition study suggested the specific presence of extracellular thrombin at least plays a partial role. Previous studies have indicated that the cultured B16F10 cell surface is a potential platform for the assembly of the prothrombinase complex, while this activity is limited to the presence of factor Xa^60^. As a large proportion of microaggregates were observed at the vascular proximity, vascular damage was a frequent event in the tumour. Indeed, we previously showed that these microaggregates co-localize to intratumoural fibrin clots, where vascular damages occur^18^. This microbleeding not only provides an ample source of prothrombin but also exposes other coagulation factors, including factor X and factor V, to tissue factor-mediated cleavage leading to the production of the activated factors Xa and Va, respectively. Thus, microbleeding activates the prothrombinase complex on the B16F10 surface, which rapidly catalyses the prothrombin conversion to active thrombin. Furthermore, the results of the present study showed an increase in platelet-B16F10 affinity after activation *in vitro* (Supplementary Fig. S2b), and the accumulation of the injected platelet complex onto tumour cells *in vivo* (Supplementary Fig. S6), suggesting a deposition process on tumour cells following platelet leakage and thrombin exposure within the microenvironment.

In conclusion, the current study reveals a thrombin-dependent mechanism of intratumoural platelet microaggregation and demonstrates that thrombin, apart from the conventional inhibition strategy, could be alternatively exploited using platelets as cytokine vectors for therapeutic purposes.

## Materials and Methods

### Mice

Female C57BL/6 mice were used for this study. The mice were purchased from Clea Japan (Tokyo, Japan) and maintained in a temperature-controlled, pathogen-free room. The plan of animal studies was approved by the Animal Committee of Graduate School of Medicine, Osaka University, and all animal experiments were carried out in accordance with the guidelines of Osaka University.

### Cell Culture

The B16F10 cell line was purchased from the American Type Culture Collection (ATCC). The cells were maintained in DMEM (Nacalai Tesque) supplemented with 10% FBS, 100 U/ml penicillin and 0.1 mg/ml streptomycin. Conditioned medium was prepared from medium of confluent B16F10 cell culture. Human aortic endothelial cells (HAEC) were purchased from Cambrex Bio Science. The cells were maintained in complete EBM-2 medium (Lonza) supplemented with HuMedia-EG growth factors (KURABO), containing final concentrations of 2% FBS, 10 ng/ml hEGF, 1.34 μg/ml hydrocortisone hemisuccinate, 5 ng/ml hFGF-B, 10 μg/ml heparin, 50 μg/ml gentamicin and 50 ng/ml amphotericin B.

### Platelet Complex Preparation and Stimulation

Platelets were isolated from the citrated blood of female C57BL/6 mice as modified from previously described protocols^18^. Platelet-rich-plasma (PRP) was prepared after centrifuging the collected blood twice at 200 × *g* for 3 minutes at room temperature. PRP was treated with 1 μM prostaglandin E_1_ (PGE_1_) for 5 minutes at room temperature and centrifuged at 900 × *g* for 5 minutes at room temperature to collect the platelets. In some experiments, PGE_1_-free PRP was centrifuged at 900 × *g* for 5 minutes at room temperature to prepare platelet-poor-plasma (PPP).

To prepare the platelet-IP10 complex, the washed platelets were incubated with 10 μg/ml recombinant IP10 (BioLegend) in mTyrode’s buffer at 37 °C for the indicated hours, followed by washing with mTyrode’s buffer. To evaluate IP10 incorporation, the complex was incubated on ice for 1 hour with lysis buffer (50 mM HEPES, 2 mM EDTA, 0.5% Triton X-100, 1× protease inhibitor cocktail (Roche) in mTyrode’s buffer). IP10 was quantified using ELISA (R&D Systems). In the release assay, platelets or the platelet-IP10 complex was stimulated with thrombin (GE Healthcare) in mTyrode’s buffer containing 0.5% BSA with or without 10 μg/ml anti-CD41 antibody (BioLegend) at 37 °C for 10 minutes. The supernatant was added to mTyrode’s buffer containing 1× protease inhibitor cocktail (Roche), 50 mM HEPES and 0.5% BSA, followed by IP10 or PDGF measurement using ELISA (R&D Systems).

To examine the activation capacity of cultured B16F10, 1 × 10^6^ platelets in 25 μl containing anti-CD41 and anti-CD62P or isotype controls were combined with 25 μl of buffer, 1 × 10^5^ B16F10 cells, 25 U/ml thrombin, fresh medium or conditioned medium in the presence or absence of 50 μl PPP. The mixture was incubated at 37 °C in the dark for 10 minutes followed by fixation in 0.5% PFA and 0.5% BSA. The samples were analysed using flow cytometry. To assess the fibrinogen binding capacity, 5 × 10^6^ platelets were incubated with buffer or 5 × 10^4^ B16F10 cells or 50 U/ml thrombin (GE Healthcare) in a 96-well plate in the presence of FITC-conjugated fibrinogen (Oxford Biomedical Research, FB01) at 37 °C for 30 minutes in the dark. The platelet samples were recovered after centrifugation and measured for fluorescence using a plate reader.

### Cytotoxicity and Proliferation Assay

5 × 10^4^ B16F10 cells were co-cultured with treatments for 24 hours. The cells were washed twice with PBS and assayed for viability using the MTS assay. The treatments of buffer, IP10, 1 × 10^7^ platelets, or platelet complex were pre-stimulated using 25 U/ml thrombin (GE Healthcare) or buffer at 37 °C for 10 minutes. To examine the impact of treatment on endothelial cells, 1 × 10^4^ HAEC were incubated in 120 μl of complete EBM-2 medium containing 5 U/ml thrombin with buffer, 60 ng IP10, 3.5 × 10^7^ platelets or the platelet-IP10 complex for 72 hours. Viable cells were detected using the MTS assay.

### Thrombin Activity Assay

B16F10 tumour and normal organs were excised from tumour-bearing female C57BL/6 mice and homogenized. The homogenates and plasma were assayed for thrombin activity using the SensoLyte 520 Thrombin Activity Assay Kit (AnaSpec). To examine thrombin activities of tissue sections, 5-μm sections were prepared and the area was measured using ImageJ software. The tissue sections were incubated with 50 μl of 1× thrombin substrate on-slide for 60 minutes in the dark. A 10-μl reaction mixture was diluted in 40 μl PBS and assayed for excitation/absorbance at 485/535 nm.

### Confocal Microscopy

The 5-μm sections were prepared from fixed and dehydrated tissue blocks of B16F10 or kidney. The slides were fixed with 4% PFA and blocked in 3% BSA in PBS. The tissues were stained using primary antibodies for cleaved thrombin and CD31 in 3% BSA followed by PBS washes. The slides were subsequently stained with secondary antibodies and washed prior to DAPI counterstaining. The mounted slides were examined using a confocal laser microscope (Nikon). To visualize the localization of IP10, the complex was fixed on slides containing 2% PFA and treated with or without 0.5% Triton X-100. The subsequent steps followed the described procedures, except a phalloidin counterstain was used. The following primary antibodies were used: anti-CD41 (2.5 μg/ml, MWReg30, BioLegend), anti-CD31 (1/50 dilution, ab28364, Abcam), anti-cleaved thrombin HC (1/50 dilution, sc-23335, Santa Cruz Biotechnology), and anti-IP10 (10 μg/ml, clone 134013, R&D Systems). Secondary antibodies with Alexa Fluor fluorescent labels were used at a 1/800 dilution (Life Technologies).

To visualize the granular localization of IP10 in the platelet-IP10 complex, platelets or the complex were fixed onto the slides using 4% PFA, followed by permeabilization and staining with primary antibodies in 4% BSA/PBST at 4 °C overnight. For α-granule staining, FITC-conjugated anti-PF4 antibody (Biorbyt Ltd., orb13908) and AF350-conjugated anti-IP10 antibody (Bioss, bs-1502R-AF350) were used. For dense granule staining, an unconjugated anti-CD63 antibody (Abcam, MX-49.129.5) was used, followed by secondary antibody staining. The stained slides were washed and mounted for confocal microscopy.

### Argatroban Inhibition

200 μg of argatroban or PBS was intraperitoneally injected into B16F10-bearing C57BL/6 mice daily for 8 days. On the eighth day, mice were perfused with 4% PFA and 5 μm-thick sections from frozen tumour tissues were stained for CD31 and CD41 as described. In the experiment where platelets were directly exposed to the tumour microenvironment, B16F10 tumours were pre-conditioned using either an intratumoural dose of 100 μl PBS or 100 μg of argatroban for 10 minutes. A total of 1 × 10^7^ PKH67-labelled platelets (Sigma) treated with 100 μg of argatroban or 100 μl PBS were intratumourally injected. After 15 minutes, the tumour was excised and 5 μm-thick frozen sections were prepared, followed by confocal microscopy using a 10x objective. The sizes of the largest 30 microaggregates in each field were determined using ImageJ with default boundary detection using a threshold of 20 pixels-squared.

### Co-culture of Splenocytes and Platelets

For qRT-PCR analysis, 1 × 10^6^ splenocytes were co-cultured with buffer or 1 × 10^7^ platelets in 100 μl of splenocyte medium (RPMI-1640 with 10% FBS, 57 μM 2-ME, 100 U/ml penicillin and 0.1 mg/ml streptomycin) containing 0 or 5 U/ml thrombin for 24 or 48 hours. The following primers were used: cd45: (fwd) 5′-TCAGAAAATGCAACAGTGACAA-3′ and (rev) 5′-CCAACTGACATCTTTCAGGTATGA-3′; and foxp3: (fwd) 5′-TCTGAAGGCAGAGTCAGGAGA-3′ and (rev) 5′-TCAGGAGGCCCACCAGTTACA-3′.

For flow cytometric analysis, cells were identically treated for 48 hours with thrombin as described above. Anti-CD4-PE conjugate (GK1.5) and anti-Foxp3-Alexa Fluor 647 conjugate (MF-14) were used according to the manufacturer’s instructions. FoxP3 staining was performed as described. A total of 2 × 10^4^ CD4^+^ cells were analysed.

### Animal Studies

To study the effect of the complex on tumour growth, 6 week-old female C57BL/6 mice were subcutaneously inoculated with 1 × 10^6^ B16F10 cells. After one week, when the tumour size reached an initial average of 40.7 (±1.9) mm^3^, the mice were randomized into experimental groups and received five intravenous treatments of saline, IP10, 4 × 10^7^ platelets or the platelet complex at 100 μl/dose on a tridaily (Schedule B) or daily (Schedule A) basis. The tumour volumes were calculated using the ellipsoid volume formula (volume = 1/2 × length × width^2^). The growth rate of each mouse was determined based on the linear regression of log volume against time.

To visualize the targeting of the platelet-IP10 complex, PBS, platelets or the platelet-IP10 complex was intravenously injected through the tail vein for 3 consecutive days. One hour after the last injection, the mice were euthanized, and the tumours were examined for CD41 and IP10. In another experiment studying the targeting capability of the platelet complex, IP10 was biotinylated using EZ-link sulpho-NHS-biotin (Thermo) and column-purified using an Ultracel-3K Membrane (Millipore). A single dose of biotinylated IP10 alone or in the platelet complex was administered through the tail vein. After two hours, the tumours were excised and homogenized in PBS containing 1× protease inhibitor (Roche), followed by the addition of 1% Triton X-100 and incubation at room temperature for 5 minutes. The supernatant was isolated from homogenate and assayed for total proteins using the DC Protein Assay (Bio-Rad). ELISA plates (Sumilon) were coated with 5 μg/ml anti-IP10 (clone 134013, R&D Systems) at room temperature overnight. After blocking with 1% BSA, the samples were applied and incubated at 4 °C for 48 hours. Avidin-HRP (BioLegend) was used for detection.

CO_2_ euthanasia was conducted according to the guidelines of the Animal Committee of Osaka University (Osaka, Japan).

### Flow Cytometry

Tumours were prepared using Schedule A described above. The tumours were excised after 24 hours and dissociated in 0.5% collagenase at 37 °C. The erythrocytes were lysed using HLB (IBL), and the recovered cells were subjected to antibody staining in 2% FBS for surface antigens according to the manufacturer’s instructions (BioLegend). The following antibody combinations were used: anti-CD4-FITC conjugate (GK1.5), anti-FoxP3-Alexa Fluor 647 conjugate (MF-14), and anti-CD45-PE conjugate (30-F11); anti-CD8a-FITC conjugate (53–6.7), anti-CD49b-Alexa Fluor 647 conjugate (HMα2), and anti-CD45-PE conjugate (30-F11); and anti-CD31-FITC conjugate (390), anti-CD25-Alexa Fluor 647 (PC61), and anti-CD49b-PE conjugate (DX5). For a 100-μl reaction, 0.5 μl of each antibody was used. For FoxP3 staining, the cells were treated with Cytofix/Cytoperm solution (BD Biosciences) and stained according to manufacturer’s instructions. Stained cells were resuspended in PBS with 2% FBS and analysed using a BD FACSCanto II flow cytometer (BD Biosciences). A total of 2 × 10^4^ CD45^+^ cells were analysed for CD4, CD8, and FoxP3, and a total of 1 × 10^5^ cells were analysed for CD31.

### Statistics

For experiments with an n value of more than 8, the data normality was assessed using the D’Agostino-Pearson test with an alpha level at 0.05. Normally distributed data were compared using two-tailed Student’s *t*-test, and non-normally distributed datasets were compared using the Mann-Whitney *U*-test. P-values were calculated using GraphPad. For experiments with multiple comparisons, the data normality was assessed using the Shapiro-Wilk test. One-way ANOVAs followed by Dunnett’s test or Tukey’s test were performed for data with normal distribution and equal variance. Non-normally distributed data were analysed using the Kruskal-Wallis test by rank.

## Additional Information

**How to cite this article**: Li, Y.-T. *et al.* Platelet-cytokine Complex Suppresses Tumour Growth by Exploiting Intratumoural Thrombin-dependent Platelet Aggregation. *Sci. Rep.* **6**, 25077; doi: 10.1038/srep25077 (2016).

## Supplementary Material

Supplementary Information
