# Supplementary material for: Platelet-cytokine Complex Suppresses Tumour Growth by Exploiting Intratumoural Thrombin-dependent Platelet Aggregation
Source: Sci Rep. 2016 Apr 27;6:25077. doi: 10.1038/srep25077 (PMC4846878; doi:10.1038/srep25077)
Supplement: Supplementary Information [file srep25077-s1.pdf]

Platelet-cytokine Complex Suppresses Tumor Growth by Exploiting Intratumoral Thrombin-dependent Platelet Aggregation.

Yu-Tung Li[1], Tomoyuki Nishikawa[1] and Yasufumi Kaneda[1*]

[1]Division of Gene Therapy Science, Graduate School of Medicine, Osaka University, Osaka, Japan

*Corresponding author: Yasufumi Kaneda

*Correspondence to Division of Gene Therapy Science, Graduate School of Medicine, Osaka University, 2-2 Yamada-oka, Suita, Osaka 565-0871, Japan. FAX: +81-6-6879-3909. E-mail: kaneday@gts.med.osaka-u.ac.jp

Supplementary Information

Supplementary Figure Legends

Supplementary Fig. S1. Thrombin was detected in PC3 xenograft. Sections were stained for CD31 (green) and cleaved thrombin (red) and then counterstained with DAPI. Two samples were shown. 20x objective. Scale bars, 100 µm. n=2.

Supplementary Fig. S2. B16F10 is incapable of activating platelets *in vitro*. (a) Platelets were treated with various agonists and examined for CD62P expression by flow cytometry. n=3-6. (b) Accutased B16F10 was mixed with either platelets or buffer at 37°C for 10 minutes; platelet binding was measured by flow cytometry. Independent experiments with resting (no thrombin) and activated (thrombin) platelets were shown. n=8-9. Error bars, SEM. ****p<0.001. Student's t-test. PPP: platelet-poor-plasma, FM: fresh medium, CM: conditioned medium, bound: B16F10-bound platelets. (c) Platelets were treated with buffer (0 minute) or 25 U/ml thrombin for up to 120 minutes at 37°C and then examined for activation marker expression of CD62P by flow cytometry. n=4.

Supplementary Fig. S3. Inside-out signaling marked by affinity change in fibrinogen receptor (GPIIb/IIIa) was not induced in platelets by B16F10 cells. Platelets were incubated with B16F10 cells or thrombin in the presence of FITC-conjugated fibrinogen for 30 minutes. Platelets were washed and collected followed by fluorescence measurement. P: platelet only, F10+P: platelet incubated with B16F10 cells, P+Thrombin: platelet incubated with thrombin. n=3. Error bars, SD. ****p<0.001. One-way ANOVA was followed by Dunnett's test.

Supplementary Fig. S4. IP10 in platelet-IP10 complex colocalized with PF4, an α–granular

protein. Platelet-IP10 complex or native platelets were fixed, permeabilized and stained for IP10 (blue) and PF4 (green), followed by observation with a confocal microscope. Scale bars = 25 µm.

Supplementary Fig. S5. IP10 in platelet-IP10 complex did not colocalize with CD63, a dense granule protein. Platelet-IP10 complex or native platelets were treated as described in Supplementary Fig. S4 and stained for IP10 (blue) and CD63 (red), followed by observation with a confocal microscope. Scale bars = 25 µm.

Supplementary Fig. S6. Injected platelet-IP10 complex deposited onto B16F10 cells *in vivo*. An inset of Fig. 3a showing relative positions of the injected IP10 (red) containing platelets (CD41, green) to B16F10 tumor cells (DAPI) was shown. Scale bar = 20 µm.

Supplementary Fig. S7. Injected platelet-IP10 complex co-localized with intratumoral thrombin. Platelet-IP10, platelet or PBS was intravenously injected to B16F10 xenograft bearing mice for three consecutive days. Tumors were collected one hour after the last injected and examined for thrombin (green) and IP10 (red) in the tumor were examined by confocal microscopy. Scale bar = 100 µm.

Supplementary Fig. S8. Endothelial cells were not affected by platelets and complex treatment *in vivo*. (a) HAEC was incubated with indicated treatments at 37°C for 72 hours. Cellular growth was determined by MTS assay. n=5. (b) Intratumoral cells were stained for CD31 and analyzed by flow cytometry. n=5. Error bars, SEM. ns, not significant. ****p<0.001. One-way ANOVA was followed by Dunnett’s test.

Supplementary Fig. S9. Platelet-IP10 complex was not cytotoxic to B16F10 cells. B16F10 was cultured with the indicated agonists with or without thrombin treatment. Cell viability was assessed by standard MTS assay after 24 hours. n=4. s/n: supernatant of activated platelet or platelet-IP10 complex.

Supplementary Fig. S10. Intratumoral expression of CD4 and FoxP3 on $CD45^+$ immune cells. Representative plots for intratumoral cells analyzed for CD45 (top panel), $CD45^+$cells analyzed for CD4 and FoxP3 (middle panel), $CD4^+$ cells analyzed for FoxP3 were shown for Fig. 5a - 5c.

Supplementary Fig. S11. Intratumoral expression of CD8 and CD49b on $CD45^+$ immune cells. Representative plots for CD45 (top panel) were shown for Fig. 5a. $CD8^-$ cells were analyzed for CD49b and vice versa (middle panel for CD8; bottom panels for CD49b).

Supplementary Fig. S12. Intratumoral FoxP3 expression reduction was induced by platelet-IP10 complex. Intratumoral expression of FoxP3 was analyzed by qRT-PCR 24 hours after a single intravenous dose of saline or platelet-IP10 complex. n=8-9. Error bars, SEM. *$p<0.05$. Student’s t-test.

Supplementary Fig. S13. FoxP3 expression on $CD4^+$ splenocytes. Splenocytes were co-cultured with buffer (upper panel) or activated platelets (lower panel) for 48 hours followed by flow cytometry. Representative plots for CD4 (left column) and FoxP3 (right column) were shown for Fig. 6b.

Figure S1

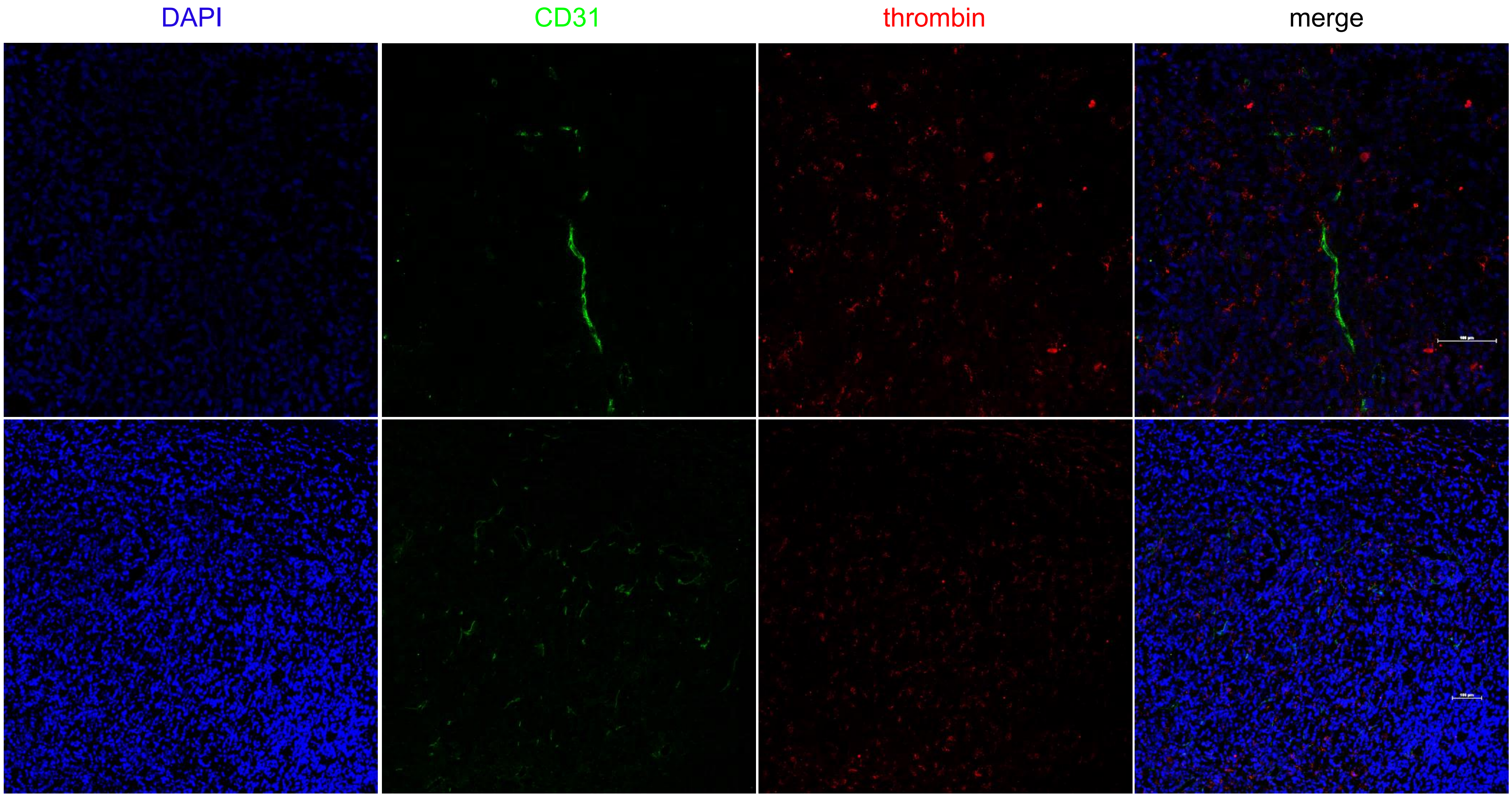

Figure S2

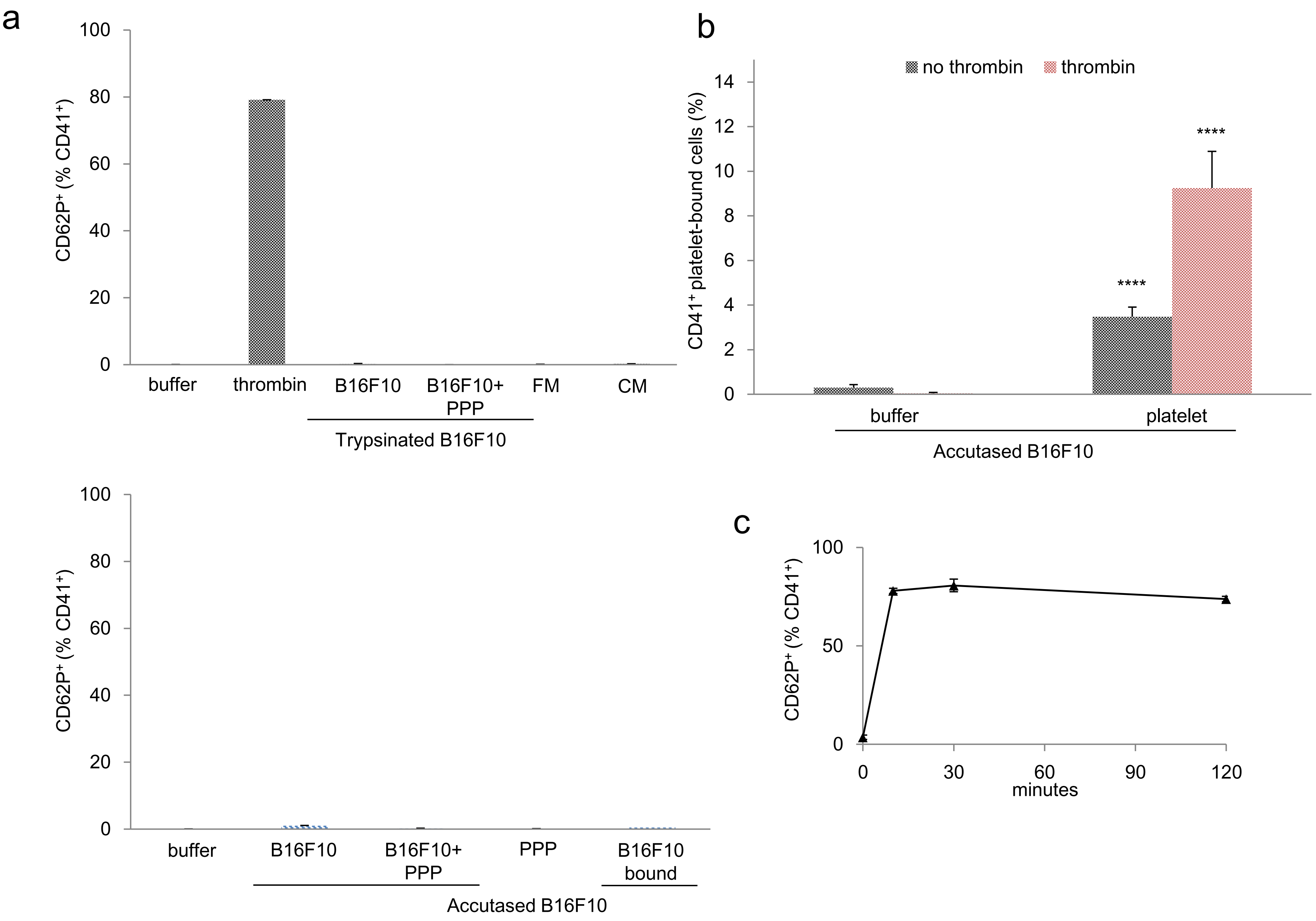
a
100
80
60
40
20
0
CD62P+ (% CD41+)
buffer
thrombin
B16F10
B16F10+
PPP
FM
CM
Trypsinated B16F10
b
no thrombin
thrombin
14
12
10
8
6
4
2
0
CD41+ platelet-bound cells (%)
****
****
buffer
platelet
Accutased B16F10
100
80
60
40
20
0
CD62P+ (% CD41+)
buffer
B16F10
B16F10+
PPP
PPP
B16F10
bound
Accutased B16F10
c
100
50
0
CD62P+ (% CD41+)
0
30
60
90
120
minutes

Figure S3

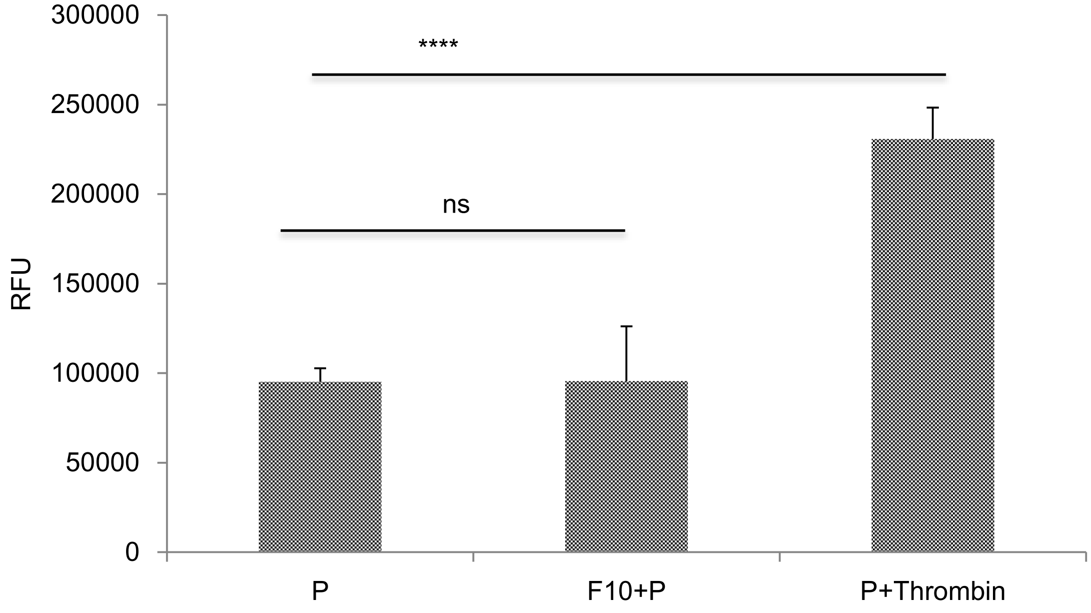
300000
250000
200000
150000
100000
50000
0
RFU
****
ns
P
F10+P
P+Thrombin

Figure S4

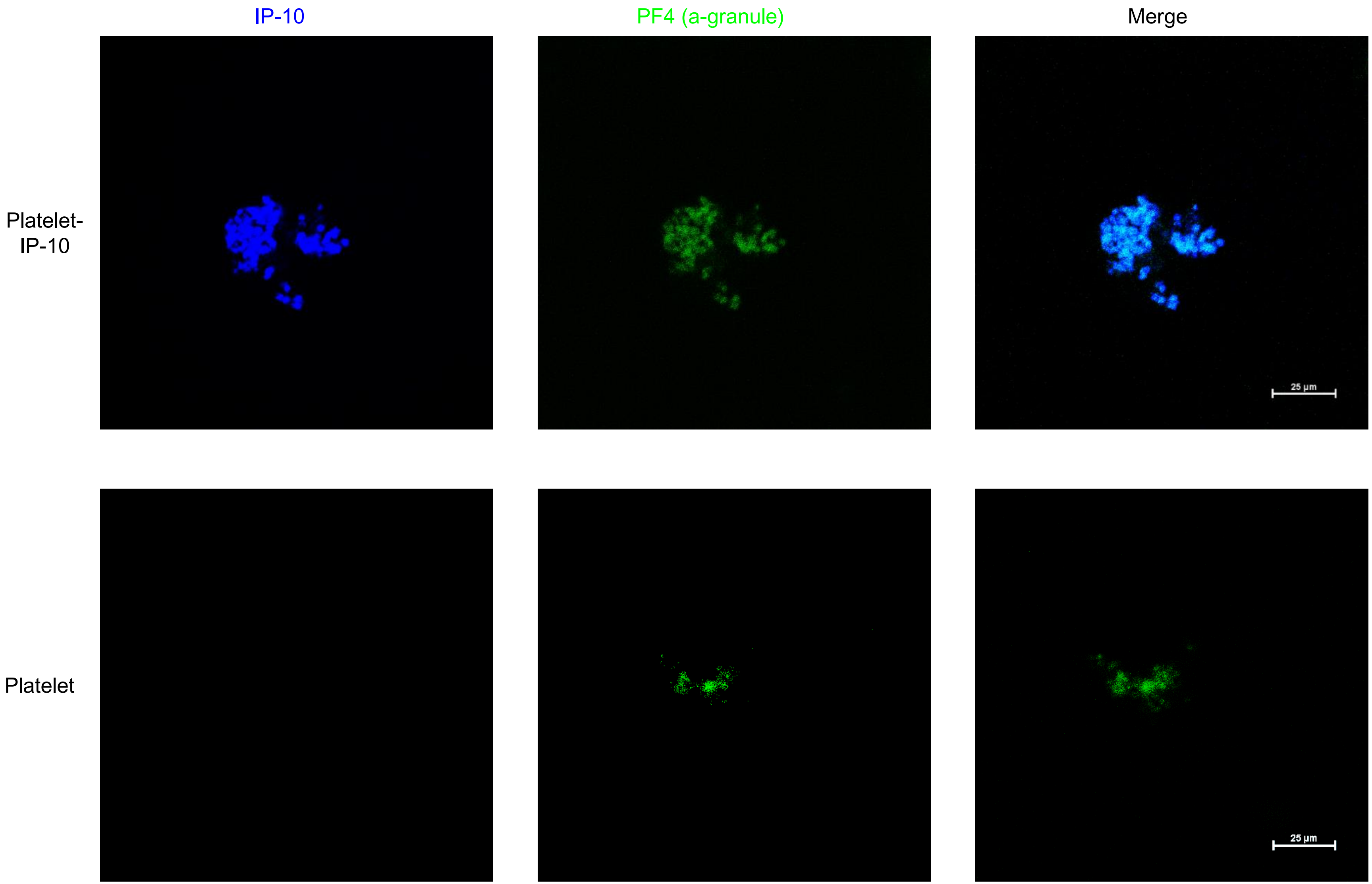

Figure S5

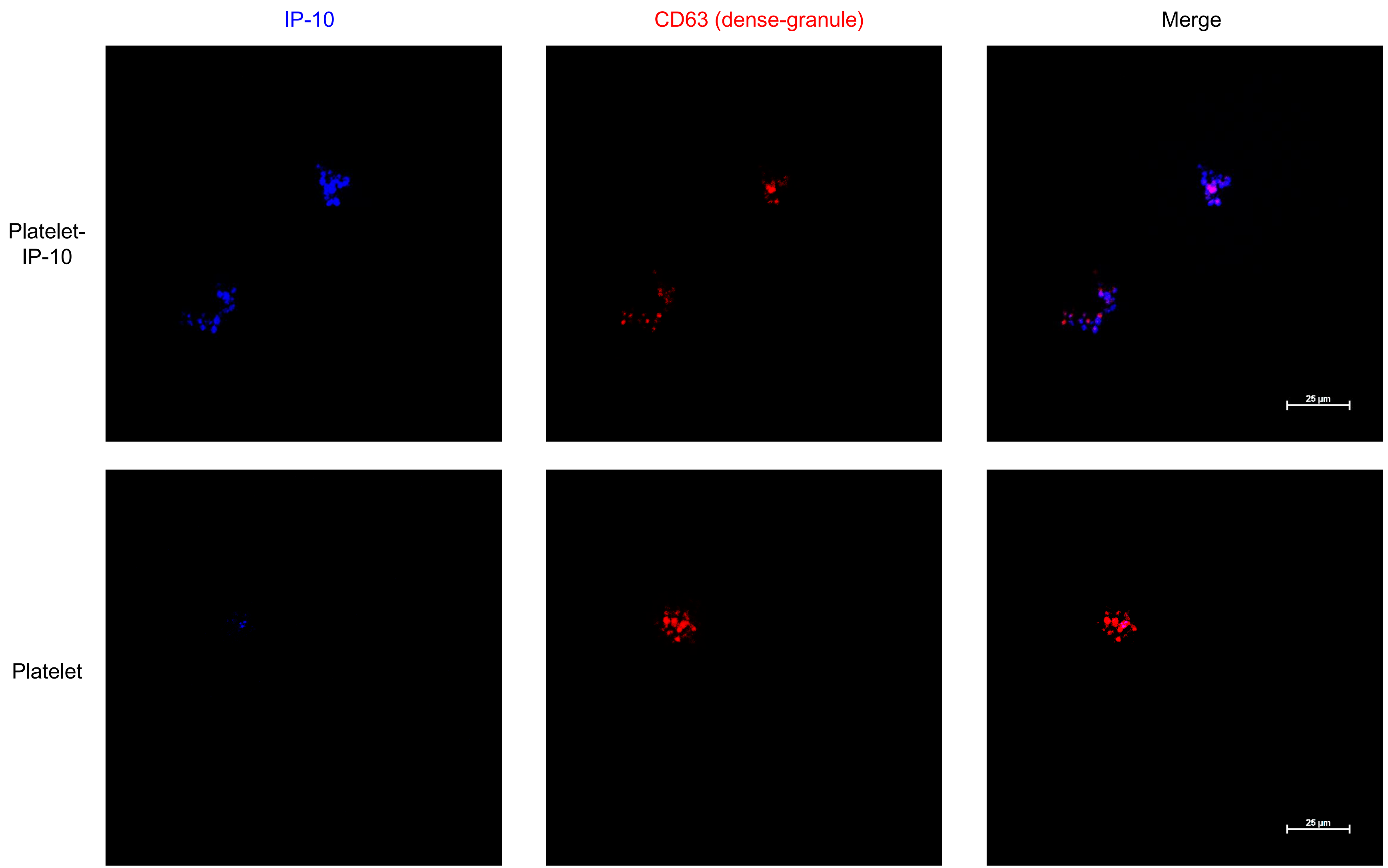
IP-10
CD63 (dense-granule)
Merge
Platelet-IP-10
Platelet
25 µm
25 µm

Figure S6

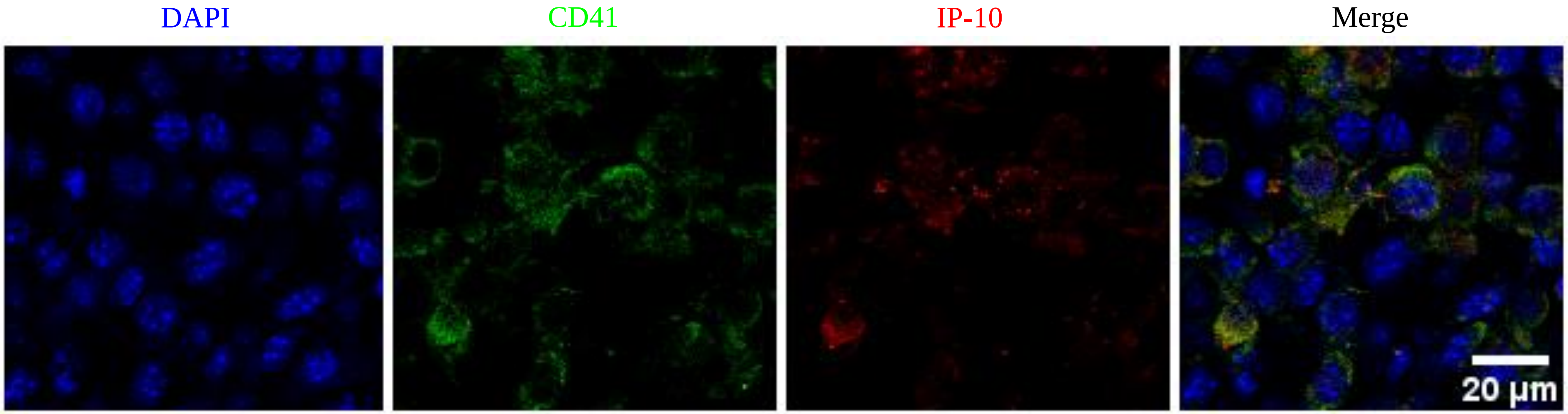

Platelet-IP10 complex

Figure S7

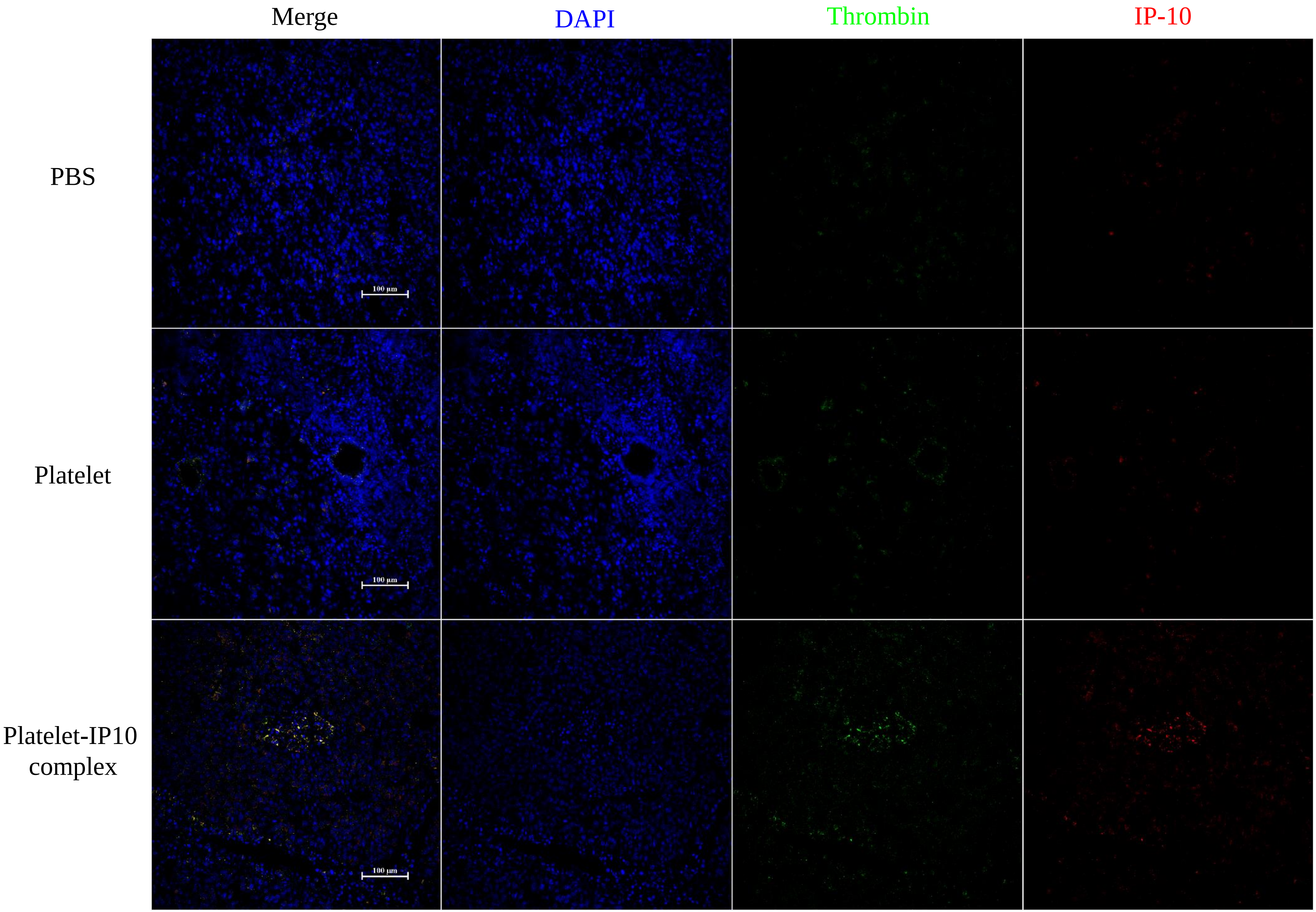

Merge
DAPI
Thrombin
IP-10
PBS
Platelet
Platelet-IP10 complex
100 μm
100 μm
100 μm

Figure S8

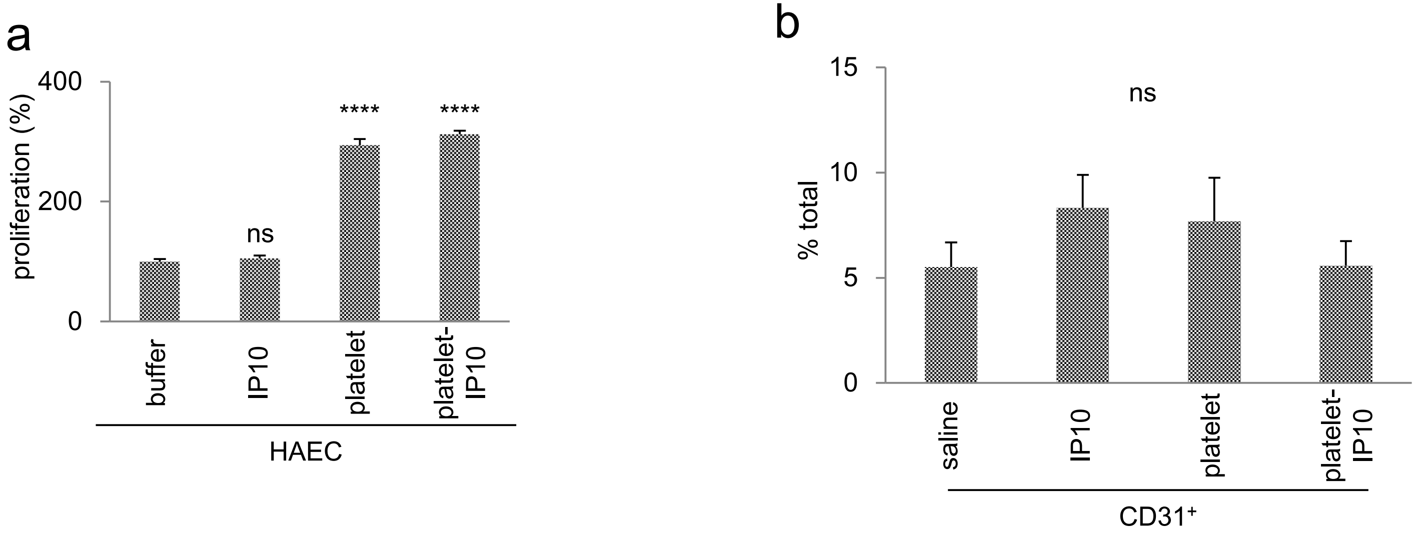

a
400
200
0
proliferation (%)
ns
****
****
buffer
IP10
platelet
platelet-IP10
HAEC
b
15
10
5
0
% total
ns
saline
IP10
platelet
platelet-IP10
CD31+

Figure S9

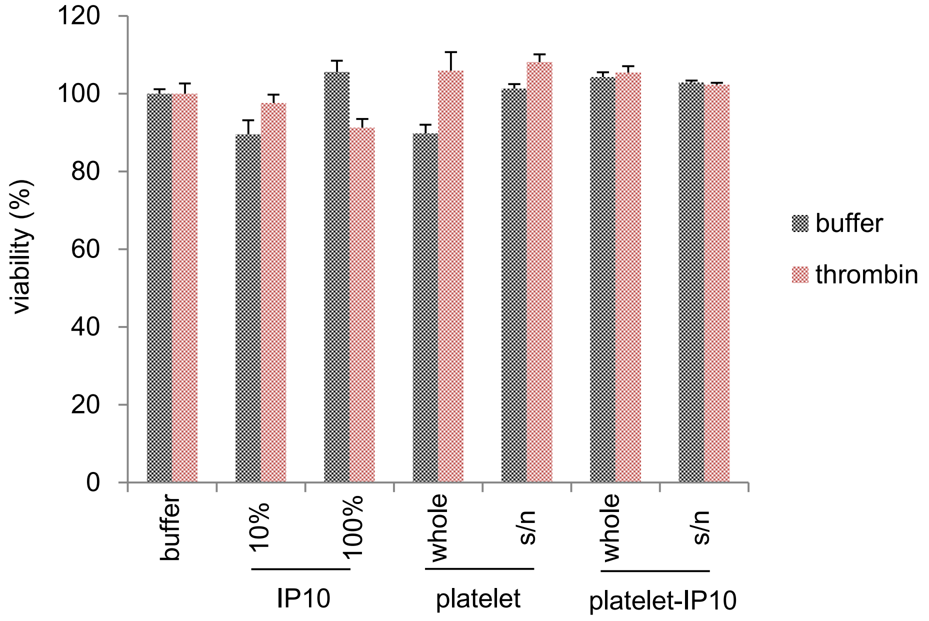

120
100
80
60
40
20
0
viability (%)
buffer
thrombin
buffer
10%
100%
IP10
whole
s/n
platelet
whole
s/n
platelet-IP10

Figure S10

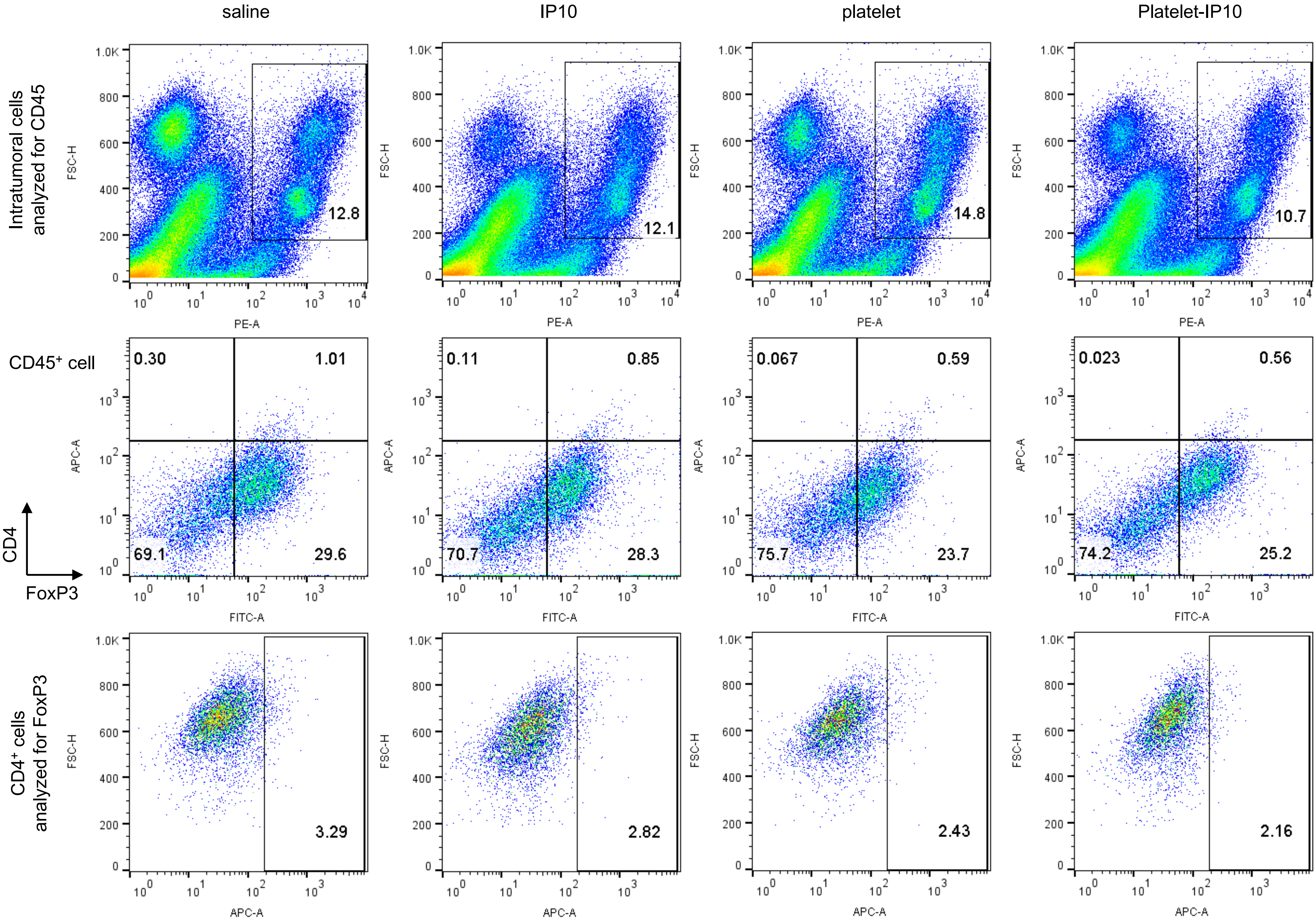

saline
IP10
platelet
Platelet-IP10
Intratumoral cells analyzed for CD45
FSC-H
PE-A
12.8
12.1
14.8
10.7
CD45⁺ cell
CD4
FoxP3
APC-A
FITC-A
0.30
1.01
69.1
29.6
0.11
0.85
70.7
28.3
0.067
0.59
75.7
23.7
0.023
0.56
74.2
25.2
CD4⁺ cells analyzed for FoxP3
3.29
2.82
2.43
2.16

Figure S11

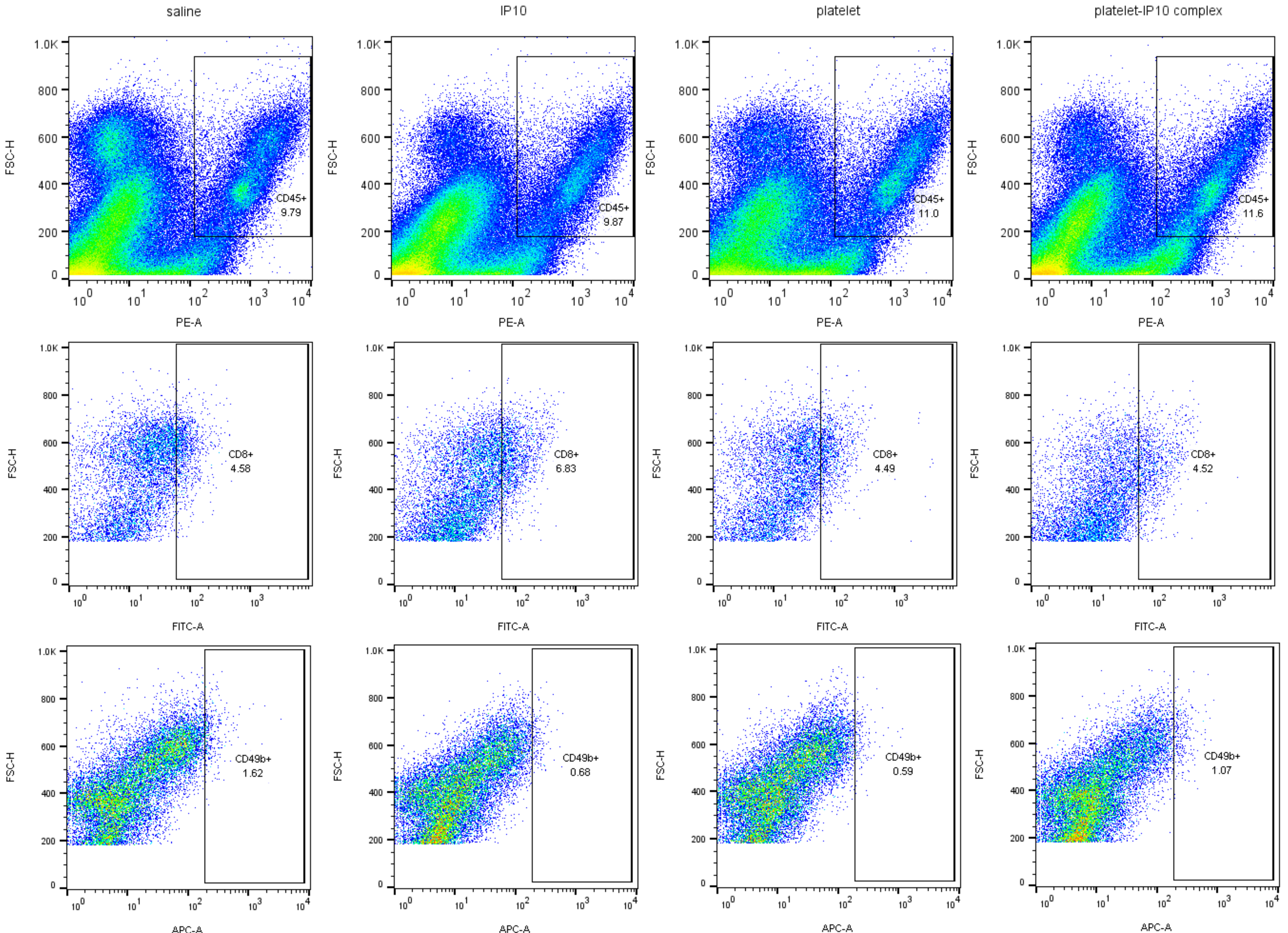

saline
IP10
platelet
platelet-IP10 complex
FSC-H
PE-A
CD45+
9.79
9.87
11.0
11.6
FITC-A
CD8+
4.58
6.83
4.49
4.52
APC-A
CD49b+
1.62
0.68
0.59
1.07

Figure S12

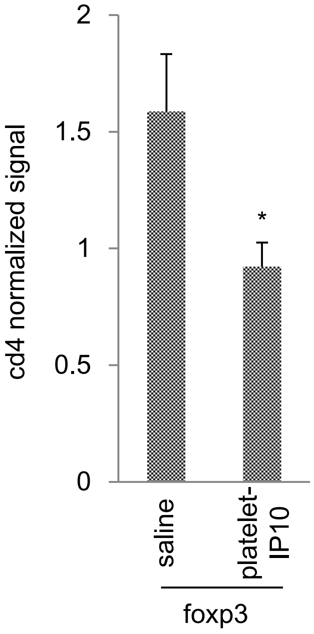

Figure S13

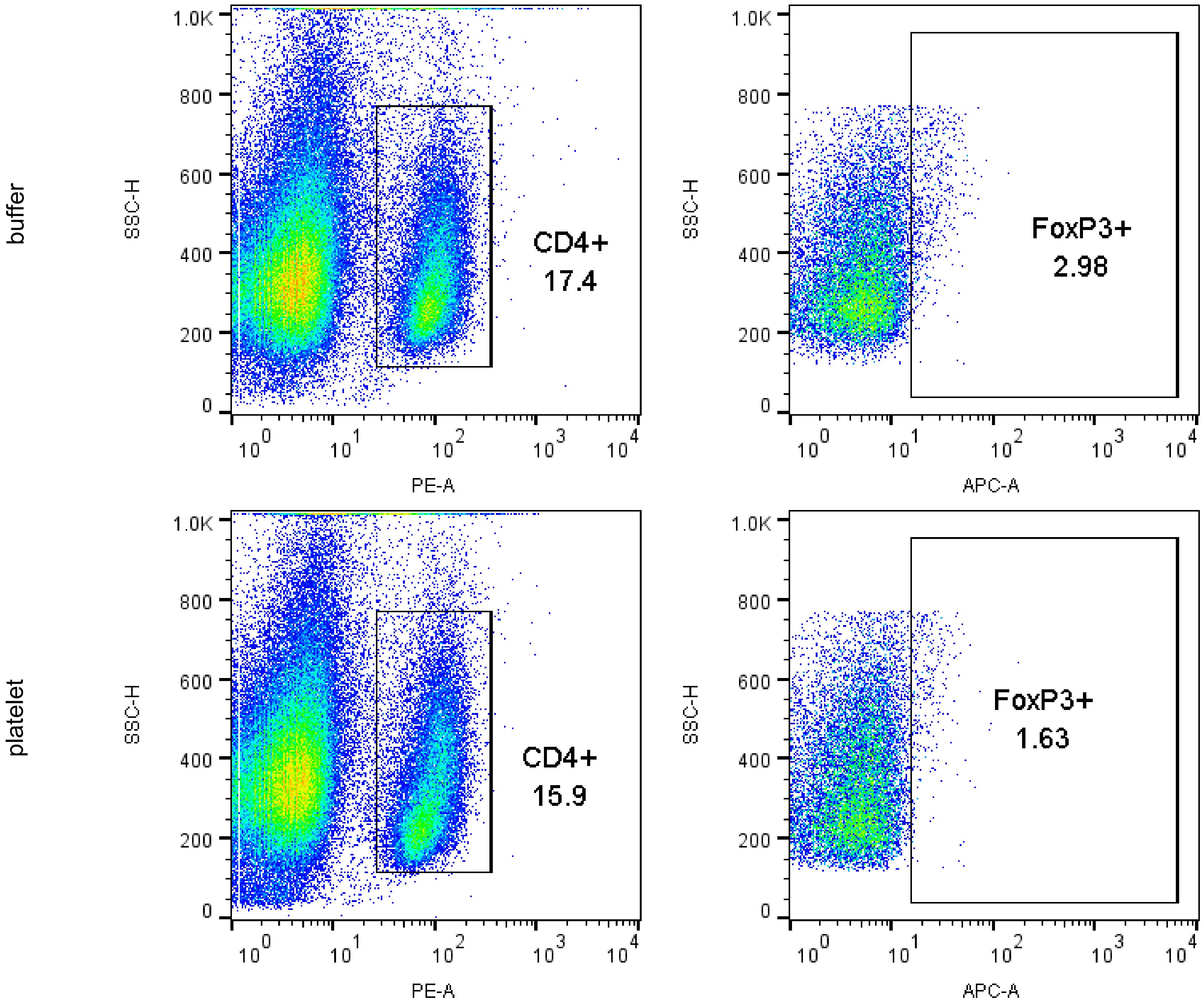

buffer
platelet
SSC-H
PE-A
APC-A
1.0K
800
600
400
200
0
10^0
10^1
10^2
10^3
10^4
CD4+
17.4
FoxP3+
2.98
CD4+
15.9
FoxP3+
1.63
